# Supplementary material for: Cell cycle-regulated transcriptional pausing of Drosophila replication-dependent histone genes
Source: bioRxiv. 2024 Dec 17:2024.12.16.628706. Preprint. [Version 1] doi: 10.1101/2024.12.16.628706 (PMC11702538; doi:10.1101/2024.12.16.628706)
Supplement: Supplement 1 [file NIHPP2024.12.16.628706v1-supplement-1.pdf]

bioRxiv preprint doi: https://doi.org/10.1101/2024.12.16.628706; this version posted December 17, 2024. The copyright holder for this preprint (which was not certified by peer review) is the author/funder, who has granted bioRxiv a license to display the preprint in perpetuity. It is made available under aCC-BY 4.0 International license.

Supplemental Figure 1. Spt6 concentrates in the HLB only during a portion of S-phase

A) Wing disc cells from 3rd instar larvae stained with anti-Mxc, anti-GFP (Spt6), and MPM-2 (P-Mxc) antibodies. Closed arrow indicates an HLB with enriched Spt6 in an S phase cell, whereas double arrow indicates an HLB lacking enriched Spt6 in an S phase cell. Open arrow indicates an HLB lacking Spt6 of a cell that is not in S phase. Scale bars are 1 μM **B-D)** Scatter plots of Mxc, Spt6, and phospho-Mxc (P-Mxc) levels within 1671 segmented HLBs from 1 wing disc as representatively shown in panel (A). Similar results were obtained from 4 independent wing discs. The values for P-Mxc (B), Spt6

bioRxiv preprint doi: https://doi.org/10.1101/2024.12.16.628706; this version posted December 17, 2024. The copyright holder for this preprint (which was not certified by peer review) is the author/funder, who has granted bioRxiv a license to display the preprint in perpetuity. It is made available under aCC-BY 4.0 International license.

(C), and Mxc (D) signals are also displayed as a heat map. Quantification of Spt6 and P-Mxc signal in segmented HLBs revealed three distinct populations of HLBs: 1) those that contain only Mxc, which are the most abundant (B, dark blue circles), 2) those that are high for both phospho-Mxc and Spt6 (C, yellow and green circles), and 3) those with high phospho-Mxc signal but low Spt6 signal (C, D).

Supplemental Figure 2.

A) Micrographs from a single representative mCherry-Rpb1 post stage 12 embryo hybridized with the CDS probe set and stained with anti-Mxc and anti-RFP antibodies. Epidermal cells arrested in $G1_{17}$ are shown in the top panels, and a section of the VNC focused on replicating neuroblasts is shown in the bottom panels. Scale bars are 3 μM. **B)** Single nuclei from regions of interest from A. Note that we are unable to detect nascent RNA in epidermal cells (top panels) but detect both nascent and cytoplasmic RNA in the neuroblast (bottom panels). Also note the appearance of Mxc staining in the neuroblast and that the hole in the “donut” shape is filled with nascent RNA signal. Scale bars are 1 μM. **C)** Micrographs from a wildtype post stage 12 embryo hybridized with both the 5’ and 3’ probe set and stained with DAPI and anti-Mxc antibody showing epidermal cells arrested in $G1_{17}$. These cells do not contain paused or elongating transcripts. Scale bars are 2 μM.

Supplemental Figure 3.

A) Cartoon showing the coverage of the three RNA fish probe sets (CDS, 5’ and 3’) in all core replication dependent histone genes. Inset shows a zoomed in example of coverage for the *H3* gene showing the relative binding locations of individual probes from the different probe sets. Made with BioRender. **B)** Table outlining the probe lengths and number of probes per gene for each probe set. **C)** Table demonstrating the coverage and sequences used for each histone gene in the 5’ and 3’ FISH sets.

Supplemental Figure 4.

A) Micrograph of the anterior portion of a gastrulating embryo stained for DAPI, Mxc, and the 3’ probe set. Notice the different mitotic domains highlighting G2, S, and M phases of the cell cycle in a single plane. Scale bars are 15 μM. **B)** Zoomed in region of interest “B” from the merge panel in A. The closed arrow points to an S-phase nucleus of cycle 15 and the open arrow highlights a G2 nucleus of cycle 14. Notice we can detect nascent 3’ transcripts in both nuclei demonstrating the continued transcription of the genes in both stages of the cell cycle at this stage in development. Scale bars are 4 μM. **C)** Zoomed in region of interest “C” from the merge panel in A. The closed arrow points to a nucleus in prophase demonstrating continued transcription as the cells enter

bioRxiv preprint doi: https://doi.org/10.1101/2024.12.16.628706; this version posted December 17, 2024. The copyright holder for this preprint (which was not certified by peer review) is the author/funder, who has granted bioRxiv a license to display the preprint in perpetuity. It is made available under aCC-BY 4.0 International license.

mitosis. The open arrow points to a small Mxc focus on a metaphase chromosome, which lacks enriched FISH signal suggesting that transcription has been aborted by this point in mitosis. Scale bars are 4 μM.

Supplemental Videos 1

A) Time lapse of a GFP-MXC and mCherry-RPB1 expressing embryo through nuclear cycles 12-14. Same embryo as the micrographs from Figure 1B. GFP-MXC is cyan and mCherry-RPB1 is yellow. **B)** Same embryo as in A but only showing GFP-MXC. **C)** Same embryo as in A but only showing mCherry-RPB1.

Supplemental Videos 2

A) Time lapse of a GFP-MXC and mCherry-RPB1 expressing embryo as it enters gastrulation. Showing cells going from G2 through mitosis and into the next S-phase. Same embryo as Figure 1C. GFP-MXC is cyan and mCherry-RPB1 is yellow. **B)** Same embryo as in A but only showing GFP-MXC. **C)** Same embryo as in A but only showing mCherry-RPB1.

Supplemental Videos 3

A) Time lapse of a GFP-SPT6 and MXC-mScarlet expressing embryo through nuclear cycles 12-14. Same embryo as Figure 1D. GFP-SPT6 is cyan and MXC-mScarlet is yellow. **B)** Same embryo as in A but only showing GFP-SPT6. **C)** Same embryo as in A but only showing MXC-mScarlet.

Supplemental Videos 4

A) Time lapse of a GFP-SPT6 and mCherry-RPB1 expressing embryo through nuclear cycles 13-14. Same embryo as Figure 1E first panel. GFP-SPT6 is cyan and mCherry-RPB1 is yellow. **B)** Same embryo as in A but only showing GFP-SPT6. **C)** Same embryo as in A but only showing MXC-mScarlet.

Supplemental Videos 5

A) Time lapse of a GFP-SPT6 and mCherry-RPB1 expressing embryo through nuclear cycle 14 as the embryo enters gastrulation. Same embryo as Figure 1E second and third panels. GFP-SPT6 is cyan and mCherry-RPB1 is yellow. **B)** Same embryo as in A but only showing GFP-SPT6. **C)** Same embryo as in A but only showing MXC-mScarlet.

bioRxiv preprint doi: https://doi.org/10.1101/2024.12.16.628706; this version posted December 17, 2024. The copyright holder for this preprint (which was not certified by peer review) is the author/funder, who has granted bioRxiv a license to display the preprint in perpetuity. It is made available under aCC-BY 4.0 International license.

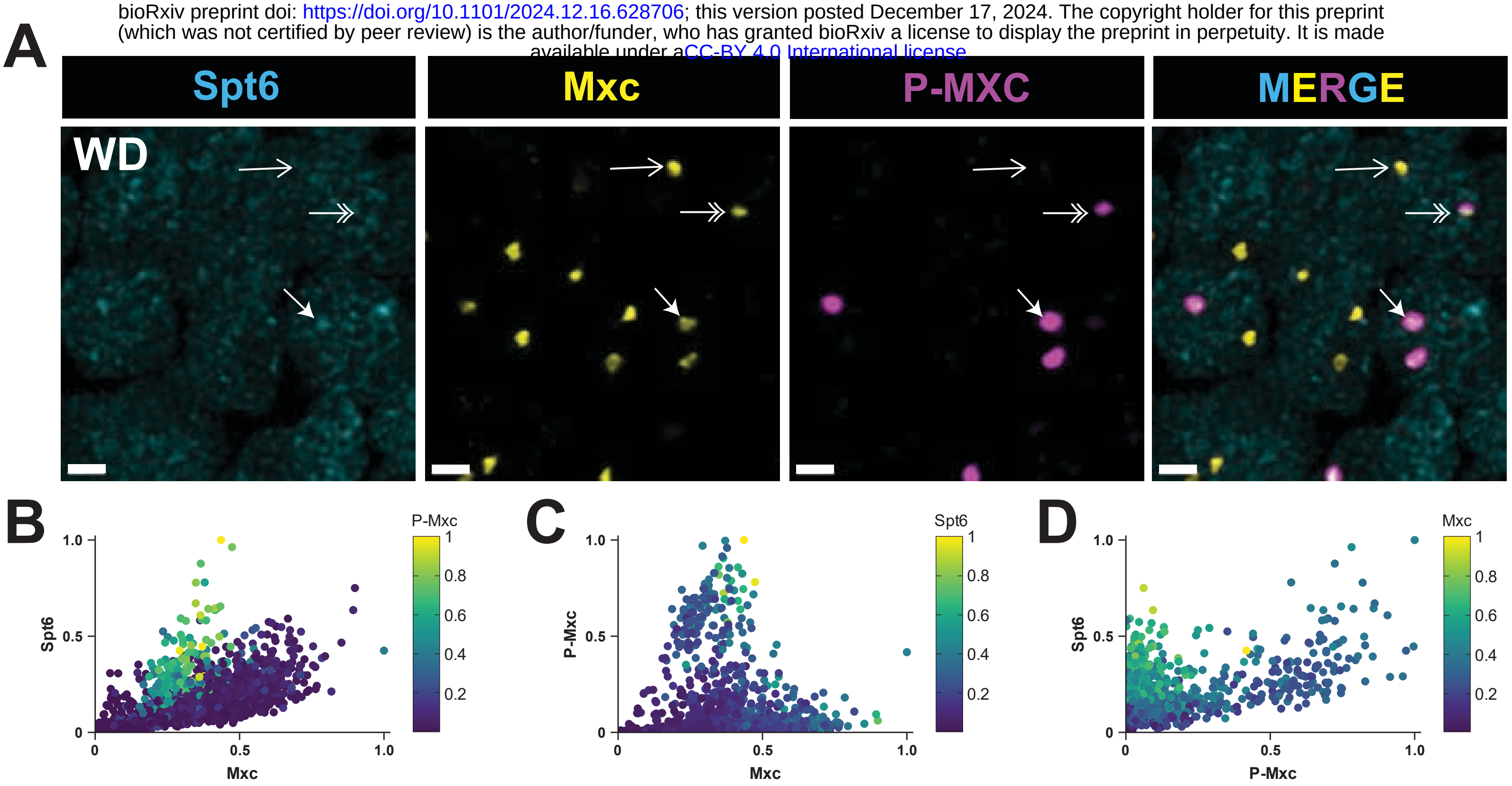

S1

bioRxiv preprint doi: https://doi.org/10.1101/2024.12.16.628706; this version posted December 17, 2024. The copyright holder for this preprint (which was not certified by peer review) is the author/funder, who has granted bioRxiv a license to display the preprint in perpetuity. It is made available under aCC-BY 4.0 International license.

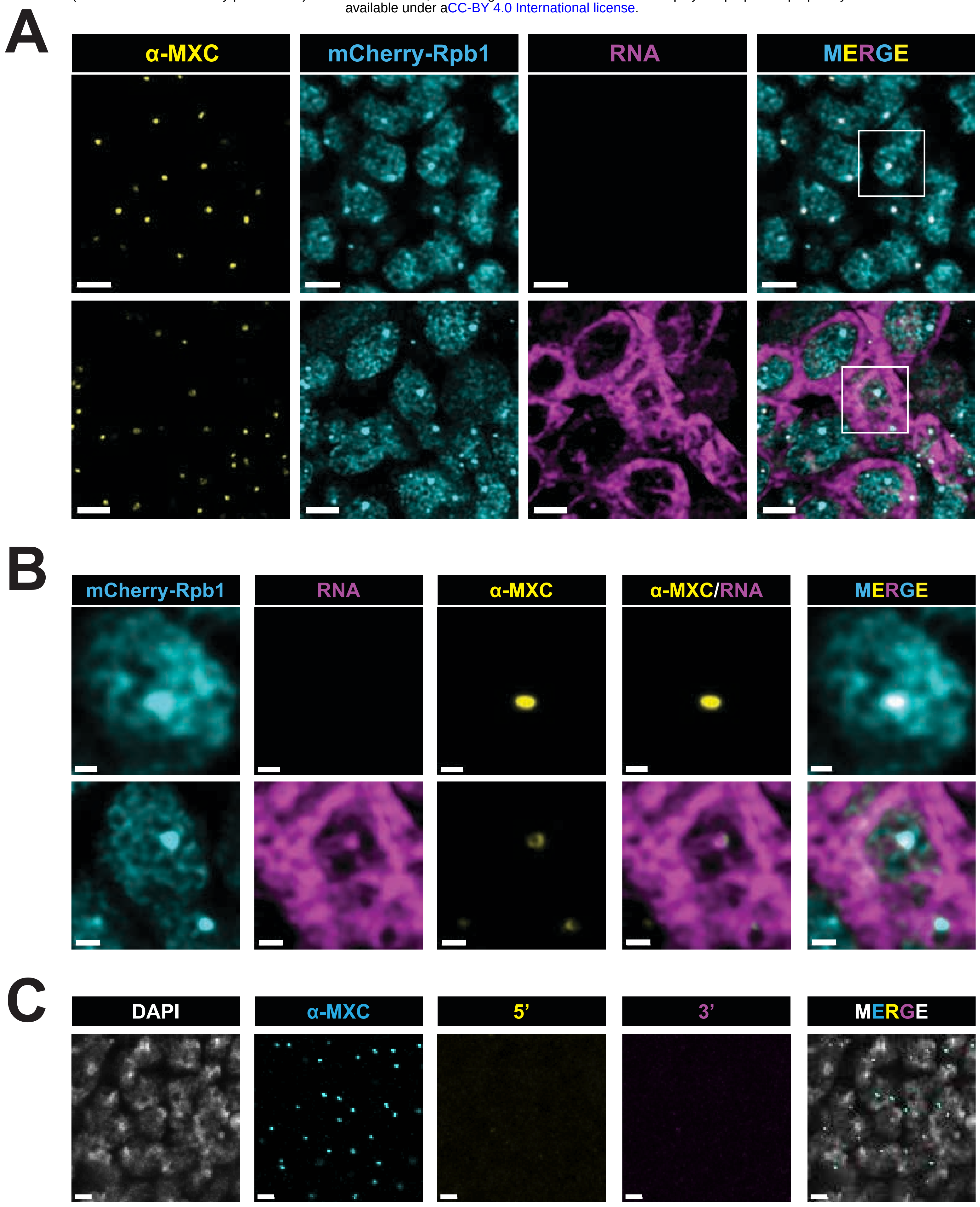

S2

bioRxiv preprint doi: https://doi.org/10.1101/2024.12.16.628706; this version posted December 17, 2024. The copyright holder for this preprint (which was not certified by peer review) is the author/funder, who has granted bioRxiv a license to display the preprint in perpetuity. It is made available under aCC-BY 4.0 International license.

A

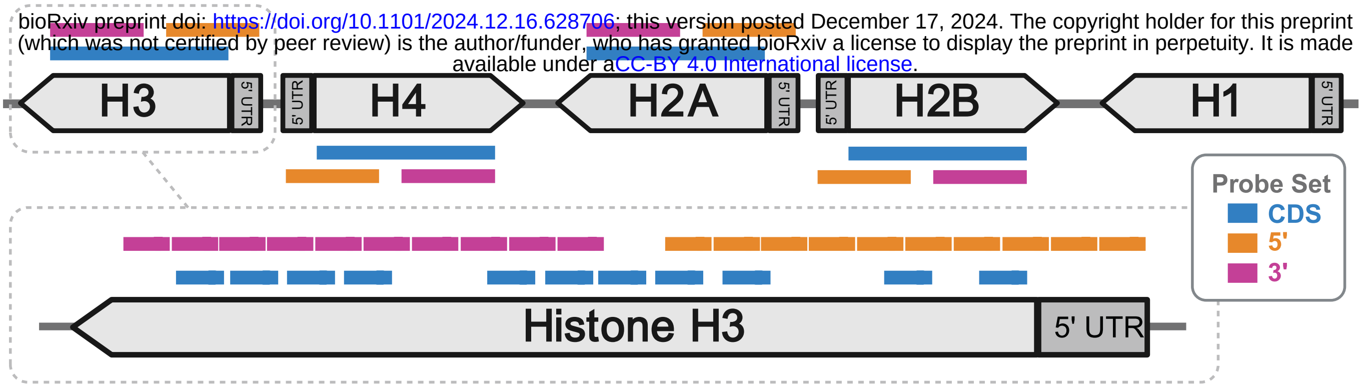

B

Probe set	Probe length (nt)	H3 probes	H4 probes	H2A probes	H2B probes	Total probes
CDS Core RD-histone	20	11	10	14	13	48
5’ Core RD-histone	18	11	10	9	10	40
3’ Core RD-histone	18	10	6	9	9	34

C

	5’ Core RD-histone			3’ Core RD-histone		
	Start (from CDS)	Stop	Coverage (bp)	Start (from CDS)	Stop	Coverage (bp)
H2A	-33	145	178	199	364	165
H2B	-31	167	198	208	370	162
H3	-48	170	218	216	397	181
H4	-51	147	198	203	311	108

S3

bioRxiv preprint doi: https://doi.org/10.1101/2024.12.16.628706; this version posted December 17, 2024. The copyright holder for this preprint
CC-BY 4.0 International license

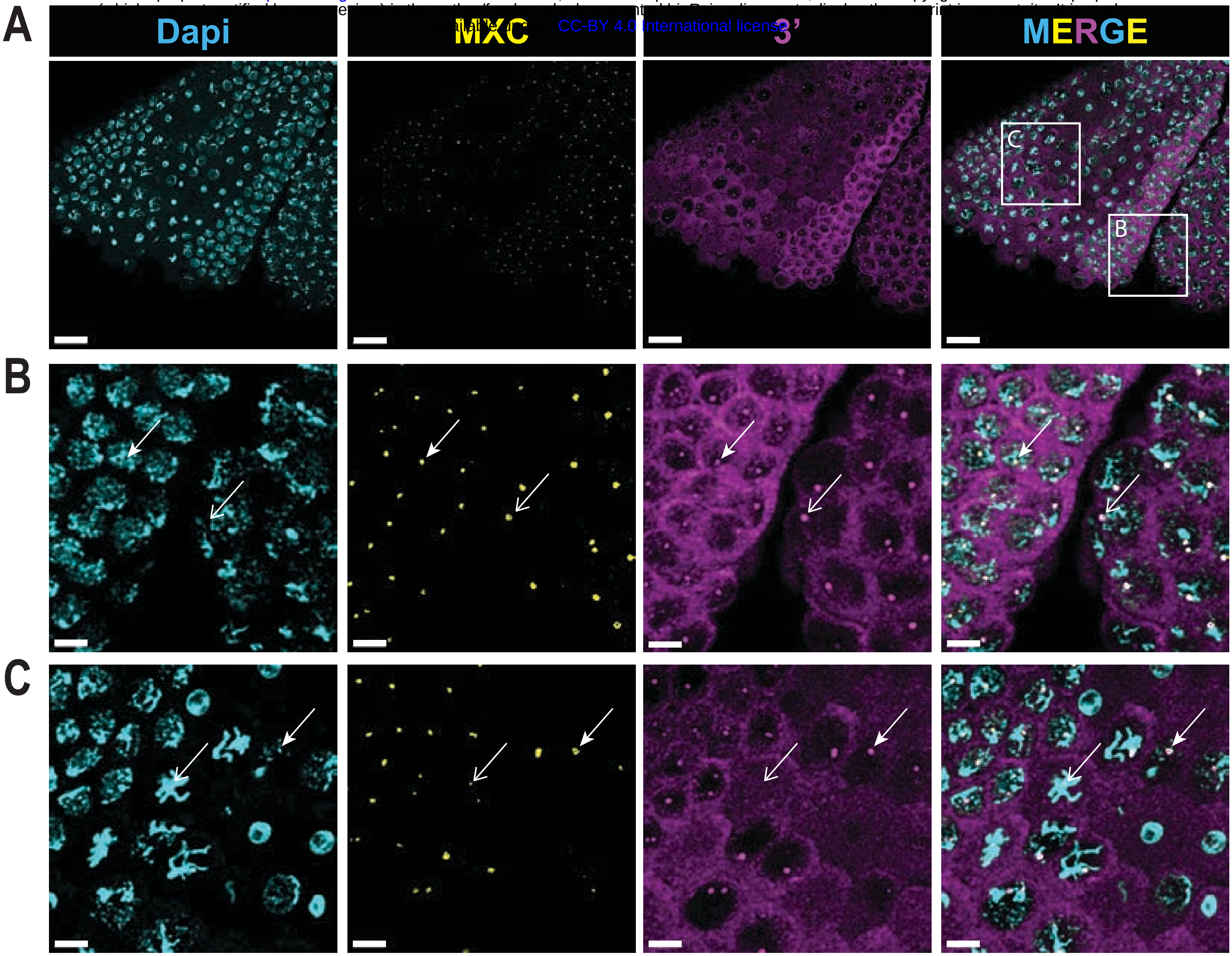

S4
